# 

**Published:** 1896-04

**Authors:** 


### The Southern Medical Record's Premium List.

Every physician sending in a subscription to **The Southern Medical Record** within 30 days, will be entitled to his choice of the following list of premiums:

1 Copy Leonard's Physician's Pocket Day Book.
1 Copy Leonard's Pocket Anatomist, Illustrated.
1 Copy Leonard's Materia Medica and The apeutics.
1 Copy Leonard's Reference and Dose Book.
1 Copy Over 1,000 Prescriptions.
1 Copy Clark's Physical Diagnosis and Uranalysis.
1 Copy Owen's Post-Mortems, How to Make.
2 Pads Gynæcological Tablets.
2 Pads Obstetric Tablets.
1 Copy Erasable Tablet Call Book.
1 Copy Fothergill's Brief Therapeutics.
1 Leonard's Flexible Uterine or Throat Applicator.
1 Hypodermic Syringe in Case.
1 Clinical Thermometer, warranted accurate.
1 Humerus (perforated metal) Splint.
4 Smith-Hodges Closed Lever Pessaries.
1 Sims' Uterine Sound.
1 Simpson's Uterine Sound.
1 Director and Tongue Tie, or Aneurism Needle.
1 Goodwillie's Nasal Speculum.
1 Hard Rubber Ear Syringe.
1 Set (four sizes) Gruber's or Toynbee's Ear Specula.
1 Dozen Surgeon's Needles, assorted sizes and kinds.
10 Patent Eye-Threading Surgeon's Needles.
1 Dozen Red Gum Catheters.
1 Olive Pointed French Catheter.
1 Double-Current Catheter, male.
1 Double-Current Catheter, female.
1 Double-Current Catheter, Lisle Thread.
1 Combined Male and Female Catheter.
3 Olive Point Lisle Catheters.
3 Olive Point Lisle Bougies.
1 Dozen Red Gum Bougies, assorted sizes.
1 Buttle's Spear-pointed Uterine Scarificator
12 Reels, Surgeon's Silk, assorted sizes.
1 Coil Silver Wire and Seven Reels Silk.
1 Dozen arbolized Sponge Tents.
3 Coils Silver Suture Wire, different sizes.
1 Vial, Twisted Silk, three sizes.
1 Vial, Braided Silk, three sizes.
1 Vial atgut (three sizes) Antiseptic Ligatures.
1 Tongue Depressor, folding, fenestrated, nickel-plated.
1 Double Canula.
1 Nasal Speculum, Goodwillie's.
1 Posterior Nasal Syringe.
Any Single-bladed, shell handled pocket instrument.
1 Gross Ear Scoop and Spoon Combined.
1 Artery Forceps, plated.
1 Dressing and Polypus Forceps, plated.
1 Pair Pocket Case Scissors, any shape.
1 Trocar, Exploring, Silver Canula.
1 Ashton's Glass Rectal Speculum.
1 Dieffenbach Self-closing Artery Clamp.
1 Soft Rubber Esmarch Bandage.
1 Gouley's Whalebone Guide.
2 Van Buren's Steel Sound.
1 Set Phalangeal (three pieces) Splints.
1 Levis's Radial splint.
1 Tibia and Fibula (perforated metal) Splint.
1 Maxilla Splint, perforated metal.
1 Femur Splint, perforated metal.
1 Long Patella Splint, perforated metal.
2 Throat Mirrors with handles.
1 Urinometer.
1 Fitche's Patent Pocket Scales.
1 Trocar (Hydrocele, etc.).
1 Ashton's Perineum Needle.
1 Exploring Needle, folding, shell handle.
1 Seaton Needle, folding, shell handle.
1 Lancet and Vaccine omb combined.
1 Bristle Probang for Throat.
1 Otis Flexible Prostatic Guide.
1 Pair Retractors, Park's or Mott's.
1 Metacarpal Saw, ebony handle.
1 Nelaton's Probe, porcelain head.
1 Powder Insufflator, with patent scoop.
1 Wild's Angular Ear Forceps.
1 Dozen Sea-Tangle Tents.
¾ Dozen Sea-Tangle Hollow Tents.
1 Dozen Slippery Elm Tents.
1 DeVilbi's Powder Blower.
1 Stylographic Pen.
1 Double Glass Magnifier.
1 Eye Scissors.
1 Dozen Surgeon's Sponges in Glass.
1 Three-blade Ivory Handle Pocketknife,
1 Pair Silver P C. Probes.
4 Hypodermic Needles.
1 Thomas' Dull Curette.
1 Sponge Holder.
1 Bone Chisel, Oval or Flat.
1 Household Syringe, H. R. Tips.
4 Retroversion Pessaries.
4 Spiral Ring S. R. Pessaries.
2 Glass Vaginal Specula.
1 Reversible Trocar.
1 Fountain Pen.

The following circular is issued by Secretary of the American Medical Association, which we print in full:

AMERICAN MEDICAL ASSOCIATION—ANNUAL ANNOUNCEMENT.

OFFICE OF THE PERMANENT SECRETARY, 1400 PINE ST., PHILADELPHIA.

The forty-seventh annual session will be held in Atlanta, Ga , on Tuesday, Wednesday, Thursday and Friday, May 5, 6, 7 and 8, commencing on Tuesday at 10 a. m.

"The delegates shall receive their appointment from permanently organized State medical societies, and such county and district medical societies *as are recognized by representation in their respective State societies,* and from the medical department of the Army and Navy, and the Marine Hospital Service of the United States.

"Each State, county and district medical society entitled to representation shall have the privilege of sending to the Association one delegate for every ten of its regular resident members, and one for every additional fraction of more than half that number: *Provided,* however, that the number of delegates for any particular State, Territory, county, city or town, shall not exceed the ratio of one in ten of the resident physicians who may have signed the Code of Ethics of the Association."

*Members by Application.*—Members by application shall consist of such members of the State, county and district medical societies entitled to representation in this Association as shall make application in writing to the Treasurer, and accompany said application with a certificate of good standing, signed by the president and secretary of the society of which they are members, and the amount of the annual subscription fee, $5. They shall have their names upon the roll, and have all the rights and privileges accorded to *permanent members*, and shall retain their membership upon the same terms.

The following resolution was adopted at the session of 1888:

*That in future, each delegate or permanent member shall, when he registers, also record the name of the Section, if any, that he will attend, and in which he will cast his vote for Section officers.*

Secretaries of medical societies, *as above designated*, are earnestly requested to forward, *at once*, lists of their delegates.

AMENDMENTS TO THE CONSTITUTION AND BY-LAWS,

Offered by Dr. G. H. Rohe:

After paragraph 8, of Article II, insert "Active members shall consist of such permanent members as have attended three meetings of the Association, and shall have all the privileges of delegates, so long as they conform to the regulations of the Association.

Insert in line 6 of paragraph 6, Article IV, relating to duties of office: "He shall in conjunction with the local committee of arrangements, make complete arrangements for the verification of the credentials of delegates, and for the registration of members. He shall also prepare annually an accurate list of members of the Association."

That Ordinance No. 18 be amended by substituting "Permanent Secretary" for "Committee of Arrangements."

Offered by the General Business Committee:

Article VII, insert: "Officers of Sections shall fill vacancies temporarily in their Executive Committee, to serve on the General Business Committee during the current meeting of the Association."

Addresses—Address in Surgery, Nicholas Senn, Chicago, Ill. Address in Medicine, William Osler, Baltimore, Md. Address in State Medicine, George H. Rohe, Baltimore, Md. Committee of Arrangements, W. F Westmoreland, Atlanta, Ga.

EXTRACTS FROM BY-LAWS.

"The Chairman of each Section shall prepare an address on the recent advancements in the branches belonging to his Section, including suggestions in regard to improvements in methods of work, and present the same to the Section over which he presides, on the first day of the annual meeting. The reading of such address not to occupy more than forty minutes."—*By-Laws.*

"It shall be the duty of every member of the Association who proposes to present a paper or report to any one of the Sections, to forward either the paper, or a title indicative of its contents and length (not to exceed twenty minutes in reading), to the Secretary of said Section, at least one month before the annual meeting at which the paper or report is to be read."—*By-Laws.*

4. *The Publication of Papers and Reports.*—"Every paper received by this Association and ordered to be published, and all plates or other means of illustration, shall be considered the exclusive property of the Association, and shall be published and sold for the exclusive benefit of the Association."—*By-Laws.*

### OFFICERS OF SECTIONS.

1. Practice of Medicine—Wm. E. Quine, Chicago, Ill., Chairman; DeLancey Rochester, Buffalo, N. Y., Secretary.

2. Obstetrics and Diseases of Women—J. Tabor Johnson, Washington, D. C., Chairman; Reuben Peterson, Grand Rapids, Mich., Secretary.

3. Surgery and Anatomy—C. A. Wheaton, St. Paul, Minn, Chairman; Wm. L. Estes, South Bethlehem, Pa., Secretary.

4. State Medicine—Chas. H. Shepard, Brooklyn, N. Y., Chairman; Elmer Lee, Chicago, Ill., Secretary.

5. Ophthalmology—Lucien Howe, Buffalo, N. Y., Chairman; Frank Allport, Minneapolis, Minn., Secretary.

6. Diseases of Children—A. C. Cotton, Chicago, Ill., Chairman; A. J. Work, Elkhart, Ind., Secretary.

7. Dental and Oral Surgery.—R. R. Andrews, Cambridge, Mass., Chairman; Eugene S. Talbot, Chicago, Ill., Secretary.

8. Neurology and Medical Jurisprudence—Thos. D. Crothers, Hartford, Conn., Chairman; W. J. Herdman, Ann Arbor, Mich., Secretary.

9. Dermatology and Spyhilography—L. D. Bulkley, New York, Chairman; T. C. Gilchrist, Baltimore, Md., Secretary.

10. Laryngology and Otology—G. V. Woolen, Indianapolis, Ind., Chairman; M. R. Ward, Pittsburg, Pa., Secretary.

11. Materia Medica and Pharmacy—F. E. Stewart, Detroit, Mich., Chairman; W. B. Hill, Milwaukee, Wis., Secretary.

12. Physiology and Dietetics—H. Bert Ellis, Los Angeles, Cal., Chairman; Henry Salzer, Baltimore, Md., Secretary.

WM. B. ATKINSON, Permanent Secretary.

---

The following is the preliminary programme of the Medical Association of Georgia. Forty-seventh Annual Session, Augusta, Georgia, April 15, 16 and 17, 1896.

FIRST DAY—WEDNESDAY, APRIL 15. MORNING SESSION—10 O'CLOCK.

Prayer.
Address of Welcome by ————
Response by————
President's Annual Address.
Report of Committee of Arrangements.
Report of Committee on Programme.
Filling Vacancies on Board of Censors.
Application for Membership.
The Treatment of Pneumonia, W. J. Mathews, M. D., Middleton.
Asthenopia, J. A. Shorter, M. D., Macon.

AFTERNOON SESSION—2 : 30 O'CLOCK.

Tetanus in the Negro, D. H. Howell, M. D., Atlanta.

Suprapubic Operation for Fibroids, with Report of Cases, D. D. Quillian, M. D., Athens.

Modern Treatment of Skin Diseases, Bernard Wolff, M. D., Atlanta.

SECOND DAY—THURSDAY, APRIL 16. MORNING SESSION—9 O'CLOCK.

Reading of Minutes.

Application for Membership.

Report of Board of Censors.

Report of Treasurer and Secretary.

Report of Standing and Special Committees.

Appointment of Auditing Committee.

The Anesthetic, L. B. Grandy, M. D., Atlanta.

Placenta Prævia, and Report of Cases, J. R. Shannon, M. D., Cabaniss.

The Medical Side of Appendicitis, E. H. Richardson, M. D., Atlanta.

Some Albuminuric Complications of Pregnancy, Howard J. Williams, M. D., Macon.

Nephritis of the Newborn, with Report of Three Cases, W. W. Terrell, M. D., Douglas.

AFTERNOON SESSION—2 : 30 O'CLOCK.

Pneumonia, J. L. Lovvorn, M. D., Bowden.

————————J. B. S. Holmes, M. D., Atlanta.

The After Treatment of Tracheotomy—Cases of Membranous Croup, R. M. Harbin, M. D.; Rome.

When do Adenoids and Polypi Cause Asthma and Hay Fever ? A. G. Hobbs, M. D., Atlanta.

Some Injuries of the Eyeball, S. Latimer Phillips, M. D., Savannah.

Treatment of Skin Disfigurements by Electrolysis, M. B. Hutchins, M. D., Atlanta.

THIRD DAY—FRIDAY, APRIL 17. MORNING SESSION.—9 O'CLOCK.

Reading of Minutes.

Application for Membership.

Report of Board of Censors.

What is the Best Treatment in Injuries of the Elbow Joint ? C. H. Richardson, M. D., Montezuma.

Inguinal Hernia, W. F. Westmoreland, M. D., Atlanta.

Report of a Few Interesting Surgical and Gynecological Cases, Montague L. Boyd, Savannah.

ELECTION OF OFFICERS AT 11 O'CLOCK.

———————————— F. W. McRae, M. D., Atlanta.

Glaucoma in Relation to General Practice, A. W. Stirling, M. D., Atlanta.

---

DR. J. H. ANDERSON, a well-known physician of Starrsville, Georgia, died on March 2.

